# The Influence of Supplementation with Zinc in Micro and Nano Forms on the Metabolism of Fatty Acids in Livers of Rats with Breast Cancer

**DOI:** 10.3390/nu13113821

**Published:** 2021-10-27

**Authors:** Agnieszka Stawarska, Małgorzata Czerwonka, Małgorzata Jelińska, Iga Piasecka, Barbara Bobrowska-Korczak

**Affiliations:** Department of Bromatology, Faculty of Pharmacy, Medical University of Warsaw, Banacha 1, 02–097 Warsaw, Poland; agnieszka.stawarska@wum.edu.pl (A.S.); malgorzata.czerwonka@wum.edu.pl (M.C.); malgorzata.jelinska@wum.edu.pl (M.J.); s067031@student.wum.edu.pl (I.P.)

**Keywords:** Zinc, nanoparticles, fatty acid metabolism, desaturases, HETE, HODE, cholesterol, oxysterols

## Abstract

The aim of this study was to investigate the effect of zinc supplementation (in the form of nano or microparticles) on the profile and metabolism of fatty acids in the liver microsomes of rats with induced breast cancer. The activity of desaturases (Δ5, Δ6, Δ9) and the level of cholesterol and its oxidized derivatives were measured. The aim of this study was also to determine the effect of various forms of zinc supplements on rats that were on 5-, 12- and 15-hydroxyeicosatetraenoic (5-, 12- and 15-HETE) and hydroxyoctadecadienoic (HODE) acids, and the level of prostaglandin E2 (PGE2). Female Spraque-Dawley rats (*n* = 24) were divided into 2 groups that were supplemented with zinc in the micro form (342 nm) or nano form (99 nm) particles, respectively, and a group with a standard diet (control group). All animals received 7,12-dimethylbenz[a]anthracene twice for the induction of breast cancer. Dietary nano-Zn supplementation increased vaccenic acid content (*p* = 0.032) and decreased Δ6-desaturase activity (*p* = 0.006), whereas micro-Zn increased cholesterol (*p* = 0.006), ∑COPs (total cholesterol-oxidation products) (*p* = 0.019) and PGE2 (*p* = 0.028) content. Dietary enrichment with Zn microparticles resulted in lower concentrations of the metabolites 15-, 12- and 5-HETE and HODE. Our study indicates that the effect of zinc supplementation on the metabolism of fatty acids in the liver microsomes under neoplastic conditions depends on the form in which it is administered.

## 1. Introduction

Currently, cancer is both a huge health and social problem worldwide. It is estimated to be responsible for one in six deaths worldwide [1]. In 2021, the World Health Organization (WHO) announced that the most commonly diagnosed cancer is no longer lung cancer, but breast cancer [1]. It is now diagnosed in 12% of women within a year. Over the past 20 years, the total number of breast cancers in the world has increased by almost 100%. In 2020, the disease was diagnosed in 2.3 million women [1]. Therefore, the search for effective methods of diagnosis and treatment, as well as compounds with potential anti-cancer activity, is still ongoing.

Cancer therapy faces many problems, including the lack of specificity of the drug in relation to cancer cells. This also causes cytotoxicity to normal cells. Nanotechnology offers some opportunities to overcome these limitations. The reduction in particle size is accompanied by the modification of chemical, physical and biological properties. The use of a nano scale reduces the degradation of the drug during its movement in vivo. This protects against both the biological and chemical environments. It minimizes side effects through more effective biocompatibility, increases bioavailability, allows targeted therapy and affects the increase in the amount of anticancer substances that can reach the tissues that are covered by the tumor [2,3]. All of this contributes to the effective treatment of the disease. Because cancer cells multiply and grow rapidly, their cell membranes are less tight than those of healthy cells. Therefore, nanoparticles are able to penetrate cancer cells and remain there [4,5]. The use of nanotechnology can contribute to the development of targeted therapies that are aimed at eliminating breast cancer stem cells. To date, several nanoparticle-based therapeutic systems have been described that are considered promising in cancer therapy. High hopes are pinned on the use of zinc, among others. Its effect on the risk and the course of cancer has been investigated and published in a number of works [6,7,8]. An increased concentration of zinc was detected in the cancerous tissues of the mammary gland in relation to the healthy tissues of the gland, while the level of this element in the serum was decreased. This increased zinc demand of cancer tissue may be due to the higher enzyme activity requiring this element as a cofactor [9,10]. Zinc is essential for the proper functioning of more than 300 enzymes that are involved in protein synthesis and fatty acid metabolism, including desaturases and elongases [11,12,13,14]. Until now, a correlation has been shown between serum concentrations of Zn and Fe, and the level of polyunsaturated fatty acids (PUFA) and the activity of some desaturases [12,15]. Our previous studies have shown a relationship between zinc supplementation and fatty acid metabolism in rat serum in the case of cancer [16]. In contrast, the results presented in this paper are intended to show whether, in the case of cancer, there is a correlation between dietary enrichment in zinc in various forms (nano and micro) and the composition and metabolism of fatty acids, including the activity of selected enzymes in liver tissue, which plays a key role in fatty acid metabolism.

Studies concerning the role of dietary fatty acids also attempt to elucidate the mechanisms of their action. The activity of eicosanoids that are formed in various tissues from PUFA (arachidonic acid, linoleic acid or eicosapentaenoic acid) under the influence of cyclooxygenases (COX) or lipoxygenases (LOX) is one such mechanism. Prostaglandins E2 and F2 (PGE2 and PGF2), prostacyclin I2, thromboxane A2 and leukotrienes (e.g., LTB4) are among the most studied eicosanoids. It has been observed that the concentration of PGE2 in tissues positively correlates with the dietary content of linoleic acid, which is a precursor of arachidonic acid. In the 1980s, it was noticed that other compounds that are formed by lipoxygenases from arachidonic acid—hydroxyeicosatetraenoic acids (HETE)—and from linoleic acid—hydroxyoctadecadienoic acids (HODE)—are also biologically active. They seem to be involved in various pathological processes such as inflammation, asthma, psoriasis, atherosclerosis and cancer [17,18,19]. An increased expression of 5- and 12-LOX has been observed in the cells of various types of cancer, including prostate, breast, colorectal and lung, which was associated with an increased 5- and 12-HETE synthesis [20,21]. These may stimulate cancer cell proliferation and inhibit apoptosis [22]. 12-HETE also facilitated tumor cell invasiveness, metastasis and angiogenesis [23]. 

The degree of cholesterol oxidation in animal tissues may be an indicator of oxidative stress [24], especially in the neoplastic process conditions. Oxidized cholesterol products (COPs), which have an additional hydroxyl, ketone or epoxide group within the steroid core, are formed mainly in the non-enzymatic process [25]; their content is correlated with the level of oxidative stress. Zinc significantly affects the metabolism of cholesterol, its formation from squalene and further transformations, but its influence is multidirectional. Zinc as a component of superoxide dismutase, which is the strongest antioxidant enzyme in the human body [26], should theoretically reduce cholesterol oxidation. However, the action of other enzyme systems, whose activity is zinc-dependent, may lead in a different direction [27]. Moreover, high doses of zinc may interfere with the bioavailability of other minerals that have a significant impact on cholesterol metabolism (e.g., copper) [28].

The aim of this study was to investigate whether dietary zinc enrichment (in micro and nano forms) affects fatty acid composition, enzyme activity (Δ5-, Δ6-, Δ9-desaturase), or levels of cholesterol and its oxidized derivatives in rat liver microsomes under the conditions of an existing cancer process. The effect of various forms of zinc on 5-, 12- and 15-hydroxyeicosatetraenoic (5-, 12- and 15-HETE) and hydroxyoctadecadienoic (HODE) acids in rat liver microsomes, as well as on prostaglandin E2 in rat livers, were also analyzed. 

## 2. Materials and Methods

### 2.1. Animals

The experiment was carried out on 24 Female Sprague-Dawley rats, after obtaining approval from the local ethical commission for animal experiments at the Medical University of Warsaw (no 645/2018). All of the individuals were fed in the standard way with Labofeed H fodder (Labofeed H, Żurawia 19, 89-240 Kcynia, Poland) and had unlimited access to water. The detailed composition of the feed that was used was described in the earlier publication [16]. The animals were kept in a room with controlled environmental conditions, at a constant temperature of 22 °C and with a 12-h light and dark cycle. After a 1-week adaptation period, the rats were randomly divided into three experimental groups of 8 animals each: one control group (standard diet) and two study groups that were supplemented with zinc in the form of microparticles (342 nm—micro-Zn) or nanoparticles (99 nm—nano-Zn), respectively. The protocol that describes in detail the process of zinc synthesis in the forms of nano and microparticles is described in the earlier publication [29]. Zinc suspension in water (concentration 4.6 mg/mL) was administered *via gavage* from the 40th day of life to the 20th week of life (0.4 mL daily). A 0.4 mL volume of water was administered to the control group by the same route and at the same frequency. On the 60th and 90th days of life, the rats were intragastrically given 7,12-dimethylbenz[a]anthracene (DMBA), which is a synthetic carcinogenic agent used to initiate the formation of some tumors, e.g., breast *adenocarcinoma*, as a suspension in rape-seed oil. The livers of rats that were sacrificed in the 20th week of life were collected for analyses.

### 2.2. Preparation of Microsomes from Liver 

Livers were homogenized in 0.25 M sucrose that was buffered to pH 7.4 with 16 mL of buffer per every 4 g of tissue. Subsequently, they were centrifuged at 1000 g for 10 min in order to remove cellular debris. Then, the supernatant was centrifuged at 16,000 g for 20 min. After rejecting the mitochondrial sediment, the remaining supernatant was centrifuged again, this time at 105,000 g for 60 min. The deposited microsomes were suspended in 4 mL of isolation medium. The prepared microsomal fraction was stored at −80 °C until analysis.

### 2.3. Determination of Fatty Acids Methyl Esters in Hepatic Microsomes

The analysis of the content and profile of fatty acids in rat liver microsomes was carried out using gas chromatography with a mass spectrometer (Pegasus^®^ BT, LECO Corporation, St. Joseph, MI, USA) after the conversion of the tested compounds into methyl derivatives. A 1.5 mL volume of 1 M NaOH was added to 200 μL of microsomes and then shaken. The samples were incubated at 80 °C for 15 min. After this time, 1.5 mL of 14% BF_3_ in methanol was added and the incubation was repeated (80 °C, 15 min). A 2 mL volume of saturated sodium chloride solution and 1 mL of hexane were added to the cooled sample. After centrifugation (20 °C, 3000 rpm, 5 min), the upper organic layer was collected and 1 µL was applied to the column. The chromatographic separation and MS detection were performed under the conditions analogous to those previously described [16].

### 2.4. Determination of Desaturases’ Activity

For testing, 0.2 mL of the microsomal suspension was collected and incubated in the reaction mixture. The reaction mixture consisted of the following components and their concentrations: 5 µM ATP; 0.1 µM CoA; 1.25 µM NADH; 0.5 µM nicotinamide; 2.25 µM glutathione; 5 μM MgCl_2_; 62.5 μM NaF; 200 nM linoleic acid/mL. The lipids were then extracted using the Folch method [30]. The difference in the arachidonic acid concentrations in the incubated (1.5 h, temp. 37 °C) and non-incubated samples, which was the measure of enzyme activity, was calculated indirectly because the amount that was produced in vitro remained closely correlated with, and was the measure of, enzyme activity. Desaturases (Δ6 and Δ5) are the enzymes that limit the rate of formation of arachidonic acid (AA) from linoleic acid (LA) in vitro; therefore, the amount of AA that is produced is strictly dependent on their activity. The content of fatty acids was determined by high performance liquid chromatography with UV/VIS detection, after their prior esterification [29] (Merck Hitachi apparatus, pump L-7100, UV/VIS detector L-74200, YMC-pack column ODS-am s-5 µm, temp. of columns 30 °C, wavelength *λ* = 198 nm). In each case, the liver microsomes were assessed for protein content using the Lowry method [31] and arachidonic acid (AA) concentration was expressed per 100 mg of protein.

In addition, the activity indices of the individual desaturases were determined: ∆6-desaturase (D6D), ∆5-desaturase (D5D), and ∆9-desaturase (D9D), each expressed by the ratio of the concentration of the corresponding product to the substrate:

- D6D = (γ-linolenic acid (GLA), C18:3, n-6)/(LA, C18:2, n-6)
- D5D = (AA, C20:4, n-6)/(Dihomo-γ-linolenic acid (DGLA), C20:3, n-6)
- D9D-16 = (Palmitoleic acid, C16:1, n-7)/(Palmitic acid (PA), C16:0)
- D9D-18 = (Oleic acid (OL), C18:1, n-9)/(Stearic acid, C18:0)
- D9D total = ((Palmitoleic acid, C16:1, n-7) + (OL, C18:1, n-9))/((PA, C16:0) + (Stearic acid, C18:0))

### 2.5. Prostaglandin E2 Content in Liver

The PGE2 content in rat liver was determined using the Cayman Chemical ELISA immunoenzyme assay (No. 514010) according to manufacturer’s instruction. Its level was expressed in pg/g of liver. 

### 2.6. Determination of Fatty Acid Metabolites

Fatty acid lipoxygenase metabolites were extracted from liver microsomes according to a modified method that was presented by Frohberg [32,33]. Briefly, 0.5 mL of methanol was pipetted into 0.6 mL of liver microsomes. Then, the samples were diluted with water to achieve a 10% methanol solution and then loaded onto the Backerbond C18 SPE cartridges (500 mg/3 mL, J.T. Baker, Rijstenborgherweg 20, Deventer, Holland), which had been preconditioned with methanol and water (10 mL each). The cartridges with the applied samples were washed with 2 mL of water, followed by 2 mL of 10% methanol. Subsequently, the studied substances were eluted with 100% methanol (3 × 0.5 mL), which was then evaporated under the nitrogen stream. The samples were dissolved in ethanol (100 μL).

Fatty acid metabolites were analyzed with high performance liquid chromatography (HPLC), using a Shimadzu system composed of a LC-20AD pump, DGU-20A5 degasser, UV-VIS SPD-10AV detector and CTO-10 AS VP oven. The studied compounds were separated on a Kinetex C18 column (Kinetex C18, 2.6 µm, 100 × 4.6 mm, Phenomenex, Torrance, CA, USA) and held at 35 °C with a mobile phase composed of methanol, water and acetic acid (73:27:0.01, by volume). The flow rate was 0.8 mL/min. Detection wavelength was 235 nm. The whole analysis lasted 30 min [34].

### 2.7. Determination of Squalene, Cholesterol and Cholesterol-Oxidation Products

Squalene, cholesterol, and cholesterol-oxidation products (COPs) were identified by gas chromatography with mass spectrometry (Pegasus® BT, LECO Corporation, St. Joseph, MI, USA). A volume of 100 μL of rat liver microsomes were used for the analysis. Sample preparation, chromatographic separation and MS detection conditions have been described in detail in our previous work [16].

### 2.8. Statistical Analysis

Statistical analysis was performed with Statistica 13.3 software (StatSoft, Kraszewskiego 36, Cracow, Poland). All data are presented as arithmetic mean and standard deviation. The analysis of variance (ANOVA), followed by the Tukey test (*α* ≤ 0.05), was used to compare the differences between the three groups, and to determine if the assumptions of normality and variance homogeneity were fulfilled. Kruskal-Wallis’ test, which is a non-parametric version of ANOVA, was applied with Dunn’s test as a post hoc test when data did not meet the assumptions of analysis. The results were considered statistically significant when *α* ≤ 0.05.

A cluster analysis was also performed and the results are presented as a dendrogram; The Ward agglomeration procedure and the Euclidean function of the distance was applied. The cut-off point was established at 33% of the maximum distance.

## 3. Results

### 3.1. Fatty Acids Profile in Hepatic Microsomes

Using gas chromatography with a mass spectrometer, a total of 21 fatty acids were detected and identified in the liver microsomes of the rats that were studied: seven belonging to saturated fatty acids (SFAs), five monounsaturated fatty acids (MUFAs) and nine PUFAs (Table 1). Of all the fatty acids, SFAs had the highest content and ranged from 46% in the nano-Zn group to 50% in the group of animals that were supplemented with Zn in the micro form (Table 2). Of the SFAs, palmitic acid (C16:0) and stearic acid (C18:0) were the most abundant in all of the study groups. Enrichment of the animal diet with zinc in the nano form significantly increased the stearic acid content (1110 ± 136 μg/mL) in the liver microsomes of rats in relation to the control group (790 ± 187 μg/mL), and a similar effect was observed after the addition of micro-Zn (1044 ± 242 μg/mL). In addition, in the groups of animals whose diet was enriched with Zn, the differences in C20:0 content compared to the control group showed an increase in the micro-Zn group and a decrease in the nano-Zn group. There were no differences in the total SFA content between the study groups (Table 1).

After analyzing the content of MUFAs in liver microsomes, we observed that they account for about 9–10% of the total fatty acid pool (Table 2). Oleic acid (C18:1, n-9) was the predominant acid in all the groups. There was a trend towards an increase in the content of MUFAs that was caused by the introduction of zinc into the diet, both in micro and nano forms. However, only two acids, C18:1 (n-7) and C20:1 (n-9), showed significant differences in the non-supplemented animals, with higher levels in the nano-Zn group (Table 1).

The modification of the diet did not significantly affect the PUFA content, which was between 41 and 44% of the total fatty acid pool (Table 2). Of all the PUFAs, LA (C18:2, n-6), AA (C20:4, n-6) and DHA (Docosahexaenoic acid, C22:6, n-3) were the most abundant (Table 1). No significant differences were observed in the concentrations of individual PUFAs or in the total n-3 and n-6 content between the zinc-supplemented and non-supplemented groups (Table 1). The n-6/n-3 ratios were also determined, but no statistically significant changes were observed.

In addition, the MUFA + PUFA/SFA ratio as well as the PUFA/SFA ratio and the peroxidation index (PI) were determined (Table 2). Regardless of the zinc supplementation, there was no significant effect on these parameters.

### 3.2. Analysis of Enzymes Activity

**Figure 1:**
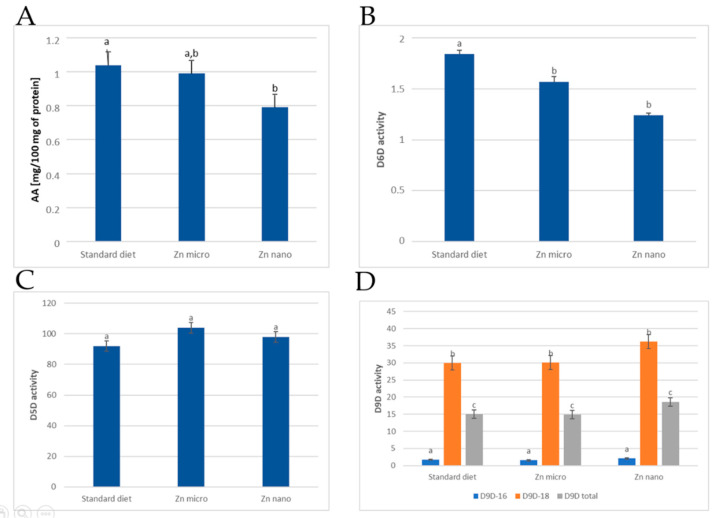
AA (arachidonic acid) concentration increase (mg/100 mg protein) (**A**). Activities of D6D (**B**), D5D (**C**), D9D-16, D9D-18, D9D-total (**D**) in hepatic microsomes of experimental groups. Standard diet—control group; micro-Zn—group receiving zinc in microparticles; nano-Zn—group receiving zinc in nanoparticles; a,b,c—homogenous groups (*α* ≤ 0.05); D6D—Δ6-desaturase index; D5D—Δ5-desaturase index; D9D-16—Δ9-desaturase index for palmitic acid, D9D-18—Δ9-desaturase index for stearic acid; D9D total—Δ9-desaturase index.

The activity of enzymes that are involved in the synthesis of arachidonic acid (D6D, D5D) was measured indirectly and expressed by the increase in arachidonic acid concentration during the application of optimal incubation conditions. In the experimental material of the animals that were supplemented with zinc nanoparticles in the diet, a significantly lower increase in AA concentration (0.79 mg/100 mg protein) was observed compared to the non-supplemented group (1.04 mg/100 mg protein) (Figure 1A). In contrast, the enrichment of the rat diet with micro-Zn did not affect the increment of AA (0.99 mg/100 mg protein) comparing to the other groups.

Desaturase indices that were expressed as the ratio of product to substrate were also determined for the following enzymes: D6D, D5D, D9D-16, D9D-18, and D9D-total. 

Dietary Zn supplementation decreased D6D activity, with lower values obtained when zinc was administered in the form of nanoparticles (Figure 1B). In contrast, there were no statistically significant differences in the effect of dietary zinc enrichment on D5D activity in any of the groups that were studied (Figure 1C).

The highest D9D activity (of all of the following: D9D-16, D9D-18, and D9D-total) was seen in the nano-Zn-supplemented group, whereas micro-Zn supplementation produced an effect comparable to the control group (Figure 1D). However, the values were not statistically significant.

### 3.3. Liver PGE2 Levels

**Figure 2:**
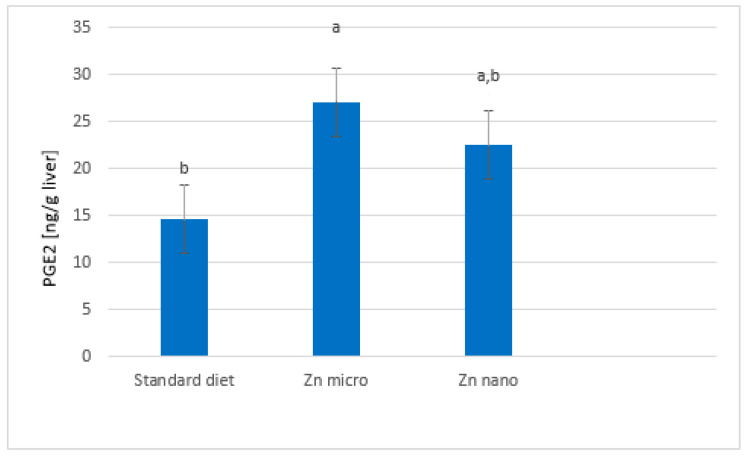
PGE2 (pg/g of liver) content in liver of experimental groups. Standard diet—control group; micro-Zn—group receiving zinc in microparticles; nano-Zn—group receiving zinc in nanoparticles. Values with a different letter index in one row are significantly different from each other (*α* ≤ 0.05) in post-hoc RIR Tukey test; PGE2—prostaglandin E2.

Based on the results presented, the highest level of PGE2 was observed in the micro-Zn group (27.08 ± 7.2 ng/g of liver), while the lowest level was observed in the non-supplemented group (14.65 ± 3.4 ng/g of liver) (*p* = 0.028) (Figure 2). Dietary zinc enrichment in the nano form increased liver PGE2 levels (22.51 ± 6.2 ng/g of liver) compared to the standard diet group, but this was not statistically significant relative to the other groups.

### 3.4. Determination of Fatty Acid Metabolites 

We planned to identify seven metabolites of fatty acids in rat liver microsomes. They are derivatives of arachidonic acid (15-HETE, 12-HETE and 5-HETE), linoleic acid (HODE) and eicosapentaenoic acid (EPA) (15-HEPE, 12-HEPE and 5-HEPE). However, EPA metabolites were undetectable, so we focused on only four compounds (HODE, 15-HETE, 12-HETE and 5-HETE).

HODE was the main eicosanoid that was detected (Table 3). Its highest concentrations were observed in the microsomes of rats that were only fed the standard diet, as well as those that were supplemented with zinc in the nano form (19.6 and 17.6 ng/mg of protein, respectively) (Table 3). These results were significantly higher compared to the group that received zinc in the micro form (7.8 ng/mg of protein). In contrast to our previous results, 12-HETE was present in the lowest amounts in liver microsomes [33]. However, similar to HODE, its content was significantly lower in the microsomes of rats that were administered zinc in the micro form (2.5 ng/mg of protein) compared to the other two groups. The 12-HETE levels were practically equal in the groups that were fed only the standard diet and supplemented with nano-zinc. A similar situation was observed for 5-HETE, whose significantly higher concentrations were detected in the microsomes of rats that were fed both the standard diet and the diet that was supplemented with nano-Zn, compared to the animals receiving zinc in the micro form (Table 3).

### 3.5. Determination of Cholesterol and Oxysterols

The content of squalene in the rat microsomes did not differ between the studied groups and the average amount, which was 9.40 µg/mL (Table 4). Cholesterol level was the highest in the group receiving zinc in microparticles and the lowest in the control group. 7-ketocholesterol (7K-Ch) was found to be the main cholesterol-oxidation product (COP); the amount of this compound was between 0.89 and 2.26 μg/mL (Table 4). In rat liver microsomes, the presence of 7α-hydroxycholesterol (7α-OH-Ch), 7β-hydroxycholesterol (7β-OH-Ch), and 5β,6β-epoxycholesterol (5,6βE-Ch) was also determined. The contents of all COPs were the highest in the group that received zinc in microparticles, which is why the sum of all the cholesterol derivatives was also the highest. The levels of these compounds in the control group and the group that received zinc in nanoparticles were similar. There were no differences between the tested groups in the COPs-to-cholesterol ratio.

**Figure 3:**
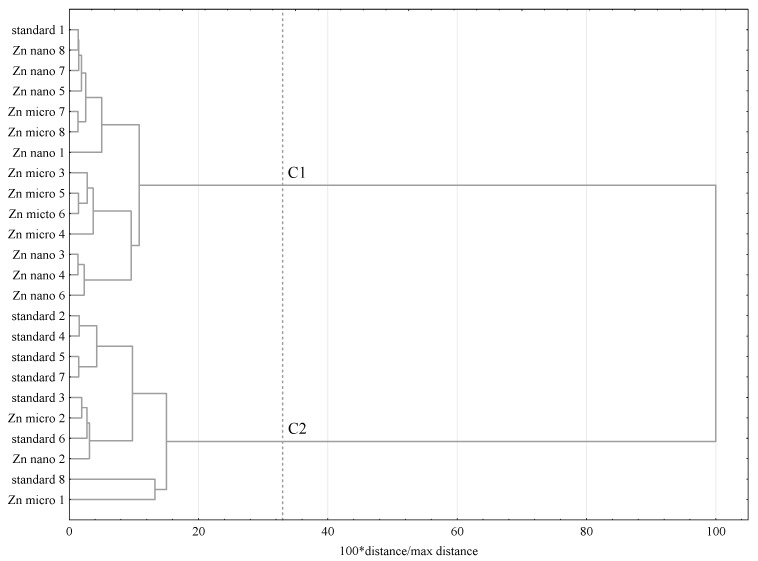
Dendrogram of similarity in lipid components’ content in rat liver microsomes; C1-C2—clusters. Standard diet—control group; micro-Zn—group receiving zinc in microparticles; nano-Zn—group receiving zinc in nanoparticles.

In the cluster analysis of the lipid components (individual fatty acids, squalene, cholesterol, COPs), two clusters were distinguished. The first cluster was predominantly (except for one) composed of rats whose diet was supplemented with zinc, whereas the second cluster was predominantly composed of rats from the control group (Figure 3).

## 4. Discussion

We have previously observed that zinc in the form of nanoparticles inhibited the development of breast tumors that were chemically induced by administering DMBA to rats [16]. Not only did we find a lower incidence of tumors in the nano-zinc-supplemented group compared to both the control group and the group that received zinc in the micro form, but there were fewer tumors per rat in the nano-Zn group and they were significantly smaller [16]. In our previous work, we noticed that zinc supplementation, in both micro and nano forms, affects the composition of fatty acids and the content of cholesterol and its derivatives in the serum of rats [16]. It also showed a significant effect of Zn on the activity of enzymes—desaturases. The present study contributes to the explanation of the mechanism of action of two forms of zinc: micro and nano. This mechanism may be connected to the activity of liver enzymes that are involved in fatty acid metabolism. The liver is the most important organ for fatty acid metabolism, and it accumulates fatty acids via their uptake from plasma by liver cells. Fatty acid synthesis and oxidation also occur here. Therefore, in the current study, we focused on investigating fatty acid metabolism in hepatic microsomes. Desaturases, whose activity is highest in the microsomal fraction, are the key enzymes that are involved in hepatic fatty acid metabolism. Their activity is important in the formation of MUFAs (D9D) and PUFAs of the n-3 and n-6 families (D6D, D5D). Moreover, PUFAs belonging to both n-3 and n-6 families compete for the same enzymes [35].

The fact that rats were chosen for this study, which focused on the effect of micro- and nano-zinc on the metabolism of fatty acids in the livers of rats with breast cancer, was not a coincidence. It is due not only to the ease of breeding, but also to the fact that the structure of their digestive system is similar to that of humans. However, the similarity in fatty acid composition in humans and rats plays the most important role in their selection. This is confirmed by Pekiner’s study [36], which showed that although the fatty acid composition of the serum and erythrocyte membranes of humans, rats, rabbits, and dogs is similar, the fatty acid content is the most quantitatively similar between humans and rats.

In our experiment, we did not observe any toxic effects of zinc in the rats. While zinc is believed to be relatively non-toxic in the range of 11–25 mg/kg of diet, it can cause copper deficiency at concentrations that exceed 250 mg/kg of diet, especially when copper levels in the diet are low [37]. On the other hand, zinc at 5 g/kg of diet inhibits animal growth and may cause anemia and/or anorexia, even with an adequate copper content in the diet. The zinc doses used in our study did not cause any toxic effects such as weight loss. 

The total content of SFAs in the rat liver microsomes did not differ between the study groups, but differences were observed in the contents of two acids, C18:0 and C20:0, with the introduction of zinc into the diet (regardless of form), resulting in an increase in their content (Table 1). The use of different supplementations did not affect the total MUFA content, although the introduction of zinc in any form resulted in an increase in the C20:1 n-9 acid, and nano-zinc also resulted in an increase in C18:1 n-7, which is vaccenic acid (Table 1). The beneficial properties of vaccenic acid in reducing the risk of breast cancer in animals may be due to its endogenous conversion to rumenic acid, which has been associated with anticancer properties [38,39]. There was no change in the activity of Δ9 desaturase, which catalyzes the conversion of palmitic acid (D9D-16) and stearic acid (D9D-18) to monounsaturated derivatives. This was reflected in the levels of C16:1 n-7 and C18:1 n-9, which were not significantly different between the test groups (Figure 1D).

The introduction of nano-zinc into the diet resulted in an increase in the total PUFA content, including the levels of acids from the n-6 family—LA, GLA and AA—and from the n-3 family—α-linolenic acid (ALA), EPA and DHA—but these differences were not statistically significant. This does not coincide with the results obtained from the serum, where analogous supplementation resulted in a decrease in total PUFA content, especially for acids belonging to the n-3 family (EPA and DHA) [16]. The relationship between dietary Zn and PUFA levels has been confirmed in several studies, but the results are not conclusive [12,14].

Zinc is an essential component of enzymes that are involved in fatty acid metabolism. It is a cofactor of desaturases D5D and D6D. In zinc deficiency, a decrease in the activity of D5D and D6D has been found, which affects the altered composition of PUFAs in plasma, liver, and skin [40]. In addition, it has been shown that the pathological condition existing in the body can modulate enzyme activity [41]. Based on the results obtained, a negative correlation is seen between zinc supplementation in nano form and enzyme activity expressed by arachidonic acid increment. It was also found that the value of the D6D index was the lowest in the group that was supplemented with nano-zinc. This can be explained by the phenomenon of competition for enzymes between the n-3 and n-6 families, which is confirmed by the higher levels of ALA, EPA and DHA [35]. Surprisingly, this was not reflected in the amount of PGE2 that was formed, which was comparable to the standard dietary group. However, it can be assumed that it was translated into a greater number of metabolites formed from EPA, which show different physiological effects than AA derivatives. In contrast, dietary zinc supplementation in the micro form, while not significantly affecting desaturase activity, resulted in a significant increase in PGE2, relative to the other groups. This may indicate an effect of zinc in this form on the activation of the cyclo-oxygenase pathway. PGE2 contributes to tumorigenesis, which includes the participation in the initiation of carcinogenesis as well as its progression. PGE2 is involved in the stimulation of cellular proliferation and angiogenesis and in the inhibition of apoptosis of potentially cancerous cells. It has been investigated that PGE2 is produced by cancerous tumors in the breast, head, neck, prostate, and colon [42].

Another of the objectives of our study was to determine the effect of dietary zinc supplementation in micro and nano forms on the content of 15-, 12- and 5-HETE, as well as HODE, in rat liver microsomes. The compounds that were studied are either metabolites of arachidonic acid (HETE) or linoleic acid (HODE). We observed significantly lower concentrations of HODE and all three HETE isomers in the microsomes that were obtained from the rats whose diets were supplemented with micro-Zn, compared to both the group that was fed only the standard diet and the group that was supplemented with Zn in the nano form. Furthermore, HODE was the main metabolite that was detected, contrary to our previous results where all the metabolites that were mentioned were analyzed in the serum [33]. There is very limited information on the effect of zinc on the biosynthesis of eicosanoids. Therefore, the present and previous studies fill this gap and shed light on this issue. The normally occurring intracellular zinc concentrations are thought to be at picomolar or nanomolar levels [43]. It was noted that elevated intracellular zinc levels activated 12-LOX in neurons, leading to increased neuronal death [43,44]. Our earlier research confirmed that zinc may stimulate 12-LOX in tissues other than neurons, resulting in an increased 12-HETE content [33]. This may provoke 12-HETE activity, such as stimulation of cell proliferation, motility and angiogenesis. The present results appear to contribute to this issue. Perhaps nano-zinc particles may more easily penetrate cells in various tissues, thereby stimulating the biosynthesis of arachidonic acid and linoleic acid metabolites. On the other hand, it is possible that supplementation with zinc in the micro form strongly activates cyclo-oxygenase, and subsequently eicosanoid production, leading to their increased metabolism, which is confirmed by the higher level of PGE2 that we determined. This could explain the significantly lower contents of HODE and 15-, 12- and 5-HETE in the liver microsomes obtained from rats that were supplemented with zinc in the micro form.

Cholesterol is a component of biological membranes and a precursor to vitamin D, steroid hormones and bile acids. Its oxidized derivatives regulate the body’s fat and carbohydrate metabolism, influence cell apoptosis and have a pro-inflammatory effect [45]. In many studies, zinc has been found to modulate cholesterol levels in the body, but its impact is multidirectional. Both a deficiency and an excessive supplementation of this ingredient can cause higher levels of cholesterol in the body [46]. In the present study, zinc supplementation showed an increase in cholesterol content in rat microsomes. Perhaps it is related to two mechanisms. First, the synthesis of HMG-CoA reductase is zinc-dependent [47], and second, this component may inhibit the bioavailability of copper [28], which is also involved in the metabolism of cholesterol. This mechanism may also explain why the highest levels of oxidized cholesterol derivatives were present in the microsomes of the rats that received zinc in microparticles, but not in the nano form. Nano-Zn may not block DMT1 [48] in enterocytes to the same degree as micro-Zn. Both zinc and copper form the prosthetic groups SOD-1 and SOD-3 [49]. Therefore, the balance between these components is extremely important. However, this is only one possible mechanism. There might be multiple factors impacting the increased level of cholesterol and its derivatives in microsomes, as a result of zinc supplementation. Higher cholesterol levels in animals resulting from zinc administration have also been shown in other studies [50].

## 5. Conclusions

Our study is the first to show that the effect of zinc supplementation under tumor process conditions on fatty acid metabolism in the liver depends on the form in which it is administered. The increase in vaccenic acid and decrease in desaturase activity when supplemented with the nano form may explain the lower incidence of cancer in this group, and may argue in favor of the use of nanoparticle supplements. In this study, we found an adverse effect of micro-Zn on the formation of pro-inflammatory PGE2, cholesterol and COPs. On the other hand, Zn administered in this form has been shown to have beneficial effects by reducing the levels of metabolites such as 15-, 12- and 5-HETE and HODE. The obtained results confirmed some of the benefits and risks of Zn supplementation, as well as indicated new mechanisms of action of this element in the metabolism of lipid compounds in the liver. However, further research on this mineral is still desirable and well-justified.

## Figures and Tables

**Table 1:** Fatty acid content in rat liver microsomes (μg/mL) (*n* = 8 per group).

	Standard Diet	Micro-Zn	Nano-Zn	*p*-Value *
**SFA**				
Lauric acid (C12:0)	1.61 ± 0.33	1.94 ± 0.15	2.28 ± 1.33	n.s.
Myristic acid (C14:0)	7.31 ± 1.97	7.04 ± 3.01	10.3 ± 5.7	n.s.
Pentadecanoic acid (C15:0)	5.94 ± 1.00	6.23 ± 1.94	7.25 ± 3.19	n.s.
Palmitic acid (C16:0)	838 ± 135	982 ± 251	1159 ± 543	n.s.
Heptadecanoic acid (C17:0)	17.1 ± 2.9	19.3 ± 2.5	18.5 ± 3.9	n.s.
Stearic acid (C18:0)	790 ± 187 ^b^	1044 ± 242 ^a,b^	1110 ± 136 ^a^	0.020
Eicosanoic acid (C20:0)	n.d. ± 0.43	0.50 ± 0.17 ^a^	0.33 ± 0.06 ^a^	<0.001
∑SFA	1660 ± 305	2062 ± 176	2309 ± 691	n.s.
**MUFA**				
9-Hexadecenoic acid (C16:1 n-9)	4.31 ± 1.35	4.54 ± 1.98	6.78 ± 3.70	n.s.
Palmitoleic acid (C16:1 n-7)	14.2 ± 6.2	15.1 ± 6.0	25.4 ± 16.2	n.s.
Oleic acid (C18:1 n-9 OL)	229 ± 86	282 ± 90	416 ± 210	n.s.
Vaccenic acid (C18:1 n-7)	46.6± 9.7 ^b^	60.4 ± 7.3 ^a,b^	77.6 ± 31.6 ^a^	0.032
Eicosenoic acid (C20:1 n-9)	0.27 ± 0.46	1.37 ± 0.28 ^a^	1.67 ± 0.58 ^a^	<0.001
∑MUFA	295 ± 102	364 ± 104	528 ± 262	n.s.
**PUFA**				
Linoleic acid (C18:2 n-6 LA)	699 ± 230	716 ± 236	1126 ± 584	n.s.
γ-Linolenic acid (C18:3 n-6 GLA)	7.02 ± 3.93	6.43 ± 4.24	15.6 ± 10.9	n.s.
α-Linolenic acid (C18:3 n-3 ALA)	19.9 ± 12.3	19.1 ± 14.0	39.1 ± 20.6	n.s.
Eicosadienoic acid (C20:2 n-6)	2.38 ± 0.86	2.12 ± 0.53	2.22 ± 0.68	n.s.
Dihomo-γ-linolenoic (C20:3 n-6 DGLA)	5.92 ± 1.21	5.66 ± 0.81	5.71 ± 2.03	n.s.
Arachidonic acid (C20:4 n-6 AA)	630 ± 206	720 ± 101	861 ± 372	n.s.
Eicosapentaenoic acid (C20:5 n-3 EPA)	9.11 ± 2.91	8.03 ± 3.15	12.9 ± 5.5	n.s.
Docosapentaenoic acid (C22:5 n-6 DPA)	15.3 ± 3.1	15.9 ± 5.3	18.5 ± 8.8	n.s.
Docosahexaenoic acid (C22:6 n-3 DHA)	187 ± 51	215 ± 39	224 ± 99	n.s.
∑PUFA	1476 ± 411	1710 ± 277	2306 ± 1081	n.s.
n-3	231 ± 55	258 ± 45	295 ± 129	n.s.
n-6	1245 ± 373	1451 ± 250	2011 ± 960	n.s.

Standard Diet—control group; Micro-Zn—group receiving zinc in microparticles; Nano-Zn—group receiving zinc in nanoparticles; SFAs—saturated fatty acids; MUFAs—monounsaturated fatty acids; PUFAs—polyunsaturated fatty acids; * one-way ANOVA (α ≤ 0.05); n.s.—no significant; ^a,b^—homogenous groups in rows; post-hoc Tukey’s test (α ≤ 0.05); n.d.—not detected.

**Table 2:** Share of the main groups of fatty acids in the total content and peroxidability and in rat liver microsomes (*n* = 8 per group).

	Standard Diet	Micro-Zn	Nano-Zn	*p*-Value *
SFA (%)	48.8 ± 4.1	50.1 ± 5.1	46.3 ± 4.7	n.s.
MUFA (%)	8.56 ± 1.97	8.74 ± 1.92	10.0 ± 1.4	n.s.
PUFA (%)	42.6 ± 3.3	41.2 ± 3.8	43.7 ± 4.0	n.s.
n-6/n-3 PUFA	5.42 ± 1.13	5.67 ± 0.89	6.80 ± 1.03	n.s.
(MUFA+PUFA)/SFA	1.06 ± 0.17	1.02 ± 0.22	1.18 ± 0.21	n.s.
PUFA/SFA	0.88 ± 0.14	0.84 ± 0.18	0.96 ± 0.18	n.s.
PI	140 ± 17	134 ± 9	128 ± 12	n.s.

Standard diet—control group; micro-Zn—group receiving zinc in microparticles; nano-Zn—group receiving zinc in nanoparticles; SFAs—saturated fatty acids; MUFAs—monounsaturated fatty acids; PUFAs—polyunsaturated fatty acids; PI—peroxidability index; * one-way ANOVA (α ≤ 0.05); n.s.—no significant.

**Table 3:** Fatty acid metabolites (ng/mg of protein) in liver microsomes obtained from rats supplemented with various forms of zinc.

	Standard Diet	Zn Micro	Zn Nano	*p-*Value *
HODE	19.6 ± 5.4 ^a^	7.8 ± 1.1	17.6 ± 6.6 ^a^	0.0017
15-HETE	6.2 ± 1.6	5.5 ± 1.8	6.3 ± 1.7	n.s.
12-HETE	4.6 ± 0.9 ^a^	2.5 ± 1.2	4.4 ± 0.9 ^a^	<0.0001
5-HETE	6.5 ± 1.8 ^a^	3.9 ± 1.4	6.1 ±1.3 ^a^	0.0045

Standard diet—control group; micro-Zn—group receiving zinc in microparticles; nano-Zn—group receiving zinc in nanoparticles; * one-way ANOVA (α ≤ 0.05); n.s.—no significant; a—homogenous groups in rows; post-hoc Tukey’s test (α ≤ 0.05).

**Table 4:** Squalene, cholesterol and cholesterol-oxidation products (COPs) content in rat liver microsomes (*n* = 8 per group).

[μg/ mL]	Standard Diet	Zn Micro	Zn Nano	*p*-Value *
Squalene	7.10 ± 4.79	10.4 ± 4.2	8.47 ± 3.44	n.s.
Cholesterol	152 ± 37 ^b^	258 ± 85 ^a^	204 ± 41 ^a,b^	0.006
7K-Ch	1.10 ± 0.58 ^a,b^	2.26 ± 1.58 ^a^	0.89 ± 0.37 ^b^	0.026
7α-OH-Ch	0.39 ± 0.17 ^a,b^	0.66 ± 0.34 ^a^	0.33 ± 0.09 ^b^	0.022
7β-OH-Ch	0.17 ± 0.09 ^a^	0.38 ± 0.24	0.17 ± 0.05 ^a^	0.018
5.6βE-Ch	0.44 ± 0.21 ^a,b^	0.81 ± 0.45 ^a^	0.38 ± 0.13 ^b^	0.020
∑COPs	2.11 ± 1.02 ^a,b^	4.10 ± 2.51 ^a^	1.77 ± 0.61 ^b^	0.019
COPs/Ch [%]	1.40 ± 0.66	1.55 ± 0.83	0.86 ± 0.22	n.s.

Standard diet—control group; micro-Zn—group receiving zinc in microparticles; nano-Zn—group receiving zinc in nanoparticles; 7K-Ch—7-ketocholesterol; 7α-OH-Ch—7α-hydroxycholesterol; 7β-OH-Ch—7β-hydroxycholesterol; 5,6βE-Ch—5β,6β-epoxycholesterol; COPs—cholesterol-oxidation products; * one-way ANOVA (*α* = 0.05); n.s.—no significant; ^a,b^—homogenous groups in rows; post-hoc Tukey’s test (*α* ≤ 0.05).
